# Genome-Wide Identification of Stress-Associated Proteins (SAPs) Encoding A20/AN1 Zinc Finger in Almond (*Prunus dulcis*) and Their Differential Expression during Fruit Development

**DOI:** 10.3390/plants11010117

**Published:** 2021-12-31

**Authors:** Sidra Fatima, Zeeshan Zafar, Alvina Gul, Muhammad Faraz Bhatti

**Affiliations:** Atta-Ur-Rahman School of Applied Bioscience, National University of Sciences and Technology, H-12, Kashmir Highway, Islamabad 44000, Pakistan; sidrafatima49032@yahoo.com (S.F.); zeeshanzafar98@hotmail.com (Z.Z.); alvina_gul@yahoo.com (A.G.)

**Keywords:** almonds, stress-associated protein (SAP), genome-wide identification, phylogenetic analysis, defense and stress responses, RNA sequence analysis, differential gene expression

## Abstract

Stress-associated proteins (SAPs) are zinc finger proteins involved in the regulation of various stresses in a variety of plant species. A total of nine *PdSAP* genes were identified in *Prunus dulcis*. Phylogenetic and synteny analyses were performed to analyze the homology and evolutionary relationship of *PdSAP* genes. The functions of *PdSAP* genes were assessed by further analyses, including *cis*-regulatory elements, gene duplication, gene ontology, gene structure, subcellular localization, and motif pattern. This study found that *PdSAP* genes were unevenly distributed on chromosomes 2, 3, 6, and 7. Phylogenetic analysis of *PdSAP* genes with *Arabidopsis thaliana* and *Oryza sativa* suggested that six subgroups have a similar pattern of AN1 and A20 domains in each subgroup. *PdSAP* genes lacked duplicated blocks. The majority of *PdSAP* genes were localized in the nucleus region. Three hormonal and five stress *cis*-regulatory elements were found in the upstream promoter region of the *PdSAP* gene family. RNA-seq analysis revealed differential gene expression of *PdSAP* genes at days 12, 17, 22, 27, 32, and 37 of fruitlet development after flowering. This study identifies the SAP genes in *P. dulcis* and also provides insights into the expression of *PdSAP* genes in abnormal fruitlets with diapause atrophic growth at various developmental stages.

## 1. Introduction

Biotic and abiotic stresses adversely affect plants’ growth and productivity [1,2]. These stresses result in great damage to global crops by reducing their average yield [3]. To eliminate the effects of biotic and abiotic stresses, plants have developed several mechanisms, including the responsive action of stress-regulating genes for the plant’s growth and maintenance [4]. Plants mediate the early stress response by the regulation of phytohormones, such as jasmonic acid (JA), abscisic acid (ABA), and salicylic acid (SA) [5,6]. Various transcription factor proteins have also been identified that play a key role in the regulation of gene expression by interacting with *cis*-regulatory promoter elements at the transcription level [7]. The DNA binding domain of transcription factors (TFs) determines the function of the transcription factors in the regulation of gene expression [8]. The binding of transcription factors with *cis*-regulatory promoter elements results in the enhanced or reduced expressions of targeted genes [9]. Transcription factors play an active role in the growth and development of plants, including hormone signaling, organ formation, secondary metabolism, and response to various stresses [10,11]. Similarly, numerous plant transcription factors also improve tolerance against abiotic stresses in plants [12].

Almond, *Prunus dulcis*, belongs to the Rosaceae family, and its seeds have commercial importance. Its cultivation is widely spread in hot climate regions [13]. Almond is one of the most cultivated trees in Spain and one of the most cultivated crops in the Mediterranean area [14]. Recently, the USA had a high production rate of almonds [15]. It is also being cultivated in other countries, including Spain, Italy, Iran, Syria, and Turkey [16]. The nutrient content is very high in almonds, including carbohydrates, amino acids, vitamins, proteins, lipids, and secondary metabolites. The seeds of almonds have concentrated energy due to their high lipid content [17].

Fruit drop is a major concern for growers of *P. dulcis*, as it occurs at three different stages of development due to various reasons [18]. Deficient pistil development leads to the first drop of malformed flowers. Unfertilized flowers are also dropped at 3–4 weeks after the bloom. The third drop or the June drop, which is basically a physiological drop, occurs at 6 or 7 weeks after the bloom [19]. Different stresses that include drought, salt, and cold greatly affect the development and yield of almonds [15]. Although almond is a drought-resistant species, irrigation is much needed to improve fruit quality and crop yield [20].

Given the role of stresses in the fruit development of *P. dulcis*, stress-associated protein (SAP) transcription factors should regulate stress responses. SAPs have been studied in various plant species, such as maize (11 genes), cucumber (12 genes), *Arabidopsis* (14 genes), rice (18 genes), cotton (37 genes), and tomato (13 genes) [3,21,22,23,24]. Stress-associated proteins (SAPs) contain one or two domains (A20 and AN1) at their N and C terminal, respectively [25]. A20 zinc finger was first identified in humans with Cys_2_/Cys_2_ finger motifs [26]. However, the AN1 zinc finger domain was identified in *Xenopus laevis*. A20 and AN1, both zinc finger domains, have key roles in the regulation of various stresses and exist in all eukaryotes, including several plant species [26].

One remarkable characteristic of plant SAPs is that they are the key regulators of plants’ biotic and abiotic stresses. Rice SAP1 (*OsSAP1*) was first identified in plants by the induction of responses against different environmental stresses, such as drought, wounding, cold, and abscisic acid (ABA) [27]. The high expression of *OsSAP1* resulted in tolerance against cold, drought, and salt stresses during different developmental stages [28]. SAP genes are also involved in the regulation of the immune system, the maintenance of plant developmental stages, and the response to phytochromes [29]. Overexpressed SAP genes of rice and other plant species have been found to induce abiotic stress tolerance against pathogens, drought, salt, temperature, and cold stresses [28,30,31]. For example, overexpression of the *OsSAP8* gene of rice induced tolerance to different stresses, such as cold, drought, and salt, in transgenic tobacco and rice during seed germination [30]. Similarly, *OsSAP1* overexpression in transgenics showed an increase in basal resistance in tobacco leaves against the bacterial pathogen *Pseudomonas syringae* [27].

Limited studies have been carried out on *P. dulcis*. SAP genes have not been identified to date. This study is based on the identification of SAP transcription factor genes in the *P. dulcis* genome and their expression in the normal and abnormal fruitlets at various growth stages. As fruitlets are dropped due to various stresses, SAP must have a pivotal role in the regulation of these stresses, as these are expressed in response to stresses. This study used RNA-seq data from another study based on the analysis of carbon signaling genes in the fruit development of *P. dulcis* at days 12, 17, 22, 27, 32, and 37 after flowering to evaluate the expression of SAP genes in normal and abnormal fruitlets [32]. This study identified nine SAP genes in almonds at the genomic level. We performed various bioinformatics analyses, such as chromosomal position, gene structure, phylogeny, and gene duplication analyses, in addition to synteny analysis, to identify SAP genes of *P. dulcis* that are homologous with those of rice and *Arabidopsis*. This study gives insights into the functional and structural characterization of the *PdSAP* gene family, along with their differential expression in the abnormally developed fruitlets with diapause atrophic growth in comparison with the normally developed fruitlets in *Prunus dulcis*.

## 2. Results

### 2.1. Identification of PdSAP Gene Family

A total of nine SAP genes were identified in almond after the removal of 100% similar sequences. A total of seven *PdSAP* gene sequences contained both AN1 and A20 domains, whereas two *PdSAP* genes contained only the zf-AN1 domain. The protein sequences containing SAP domains are provided in Data S1. SAP gene sequences were renamed from *PdSAP1* to *PdSAP9* based on their position on chromosomes 2, 3, 6, and 7. The identified SAPs of *P. dulcis*, along with their accession numbers, domains, and chromosome numbers, are presented in Table S1. The protein sequence length of the SAP genes was between 152 amino acid residues of *PdSAP3* and 292 amino acid residues of *PdSAP7*.

### 2.2. Multiple Sequence Alignment and Phylogenetic Analysis of PdSAP

**Figure 1:**
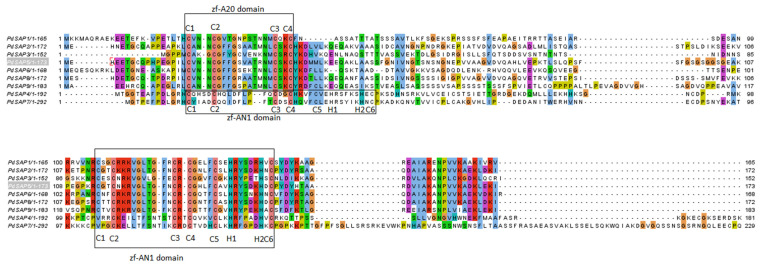
Multiple sequence alignment of SAP gene sequences. Multiple sequence alignment of zf-A20/AN1 domains using Jalview. Conserved zf-A20 and zf-AN1 domains are boxed.

**Figure 2:**
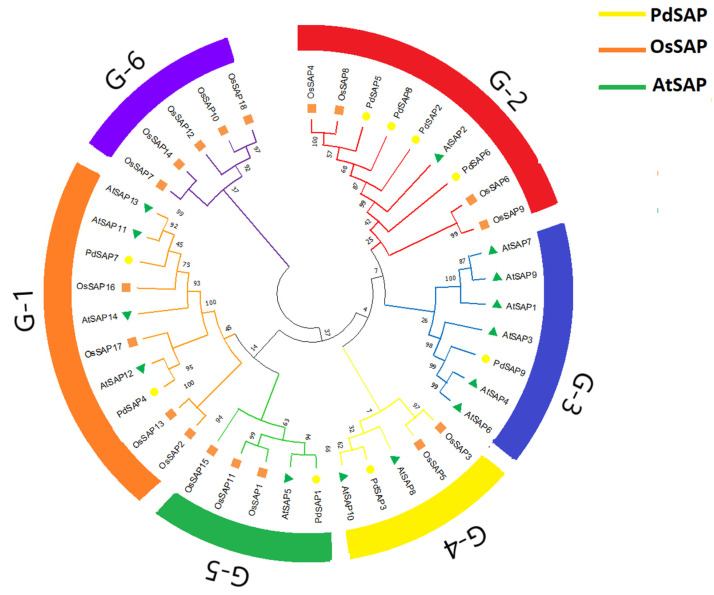
Phylogenetic tree of *PdSAP* genes with *A. thaliana* and *O. sativa. PdSAP* genes are classified into six subgroups (G1–G6) based on number of SAP genes present in groups. The six subgroups are marked in different colors.

To predict the presence of zf domains in *PdSAP*, a multiple sequence alignment of predicted protein sequences of SAP genes was carried out using MegaX-V10.2.4 software. The zinc finger domains zf-A20/A1 are highlighted in the boxes labeled in Figure 1. The peptide sequences of *AtSAP*, *PdSAP*, and *OsSAP* genes were used to construct a phylogenetic tree using the neighbor-joining method, which is shown in Figure 2. Most of the *PdSAP* genes showed close links with the *OsSAP* genes. Few genes of *PdSAP* are distantly related to *AtSAP* genes. The SAP genes of *P. dulcis*, *O. sativa*, and *A. thaliana* are highlighted in different colors. The phylogenetic tree was divided into six subgroups (G1–G6). The G-1 subgroup was the largest group, with two members of *PdSAP* (*PdSAP7* and *PdSAP4*), four members of *A. thaliana* (*AtSAP11*, *AtSAP12*, *AtSAP13*, and *AtSAP14*), and four members of *OsSAP* genes (*OsSAP2*, *OsSAP13*, *OsSAP16*, and *OsSAP17*).

Members of the G-1 subgroup mainly possessed the AN1 domain, except for *OsSAP2*, which harbored both AN1 and A20 domains. The G-2 subgroup possessed both AN1 and A20 domains with four *PdSAP* genes (*PdSAP2*, *PdSAP5*, *PdSAP6*, and *PdSAP8*), whereas the G-3 subgroup consisted of only *PdSAP9*. The G-4 subgroup and the G-5 subgroup consisted of five members with *PdSAP3* and *PdSAP1*, respectively. Members of the G-4 subgroup contained only the AN1 domain, except for *PdSAP3*. Members of the G-5 subgroup contained both A20 and AN1 domains, except for *OsSAP15*, which harbored only the AN1 domain. The G-6 subgroup showed no relationship with *PdSAP* genes; however, it only possessed *OsSAP* genes with both AN1 and A20 domains.

### 2.3. Chromosomal Position and Cis-Acting Regulatory Analysis of PdSAP Genes

**Figure 3:**
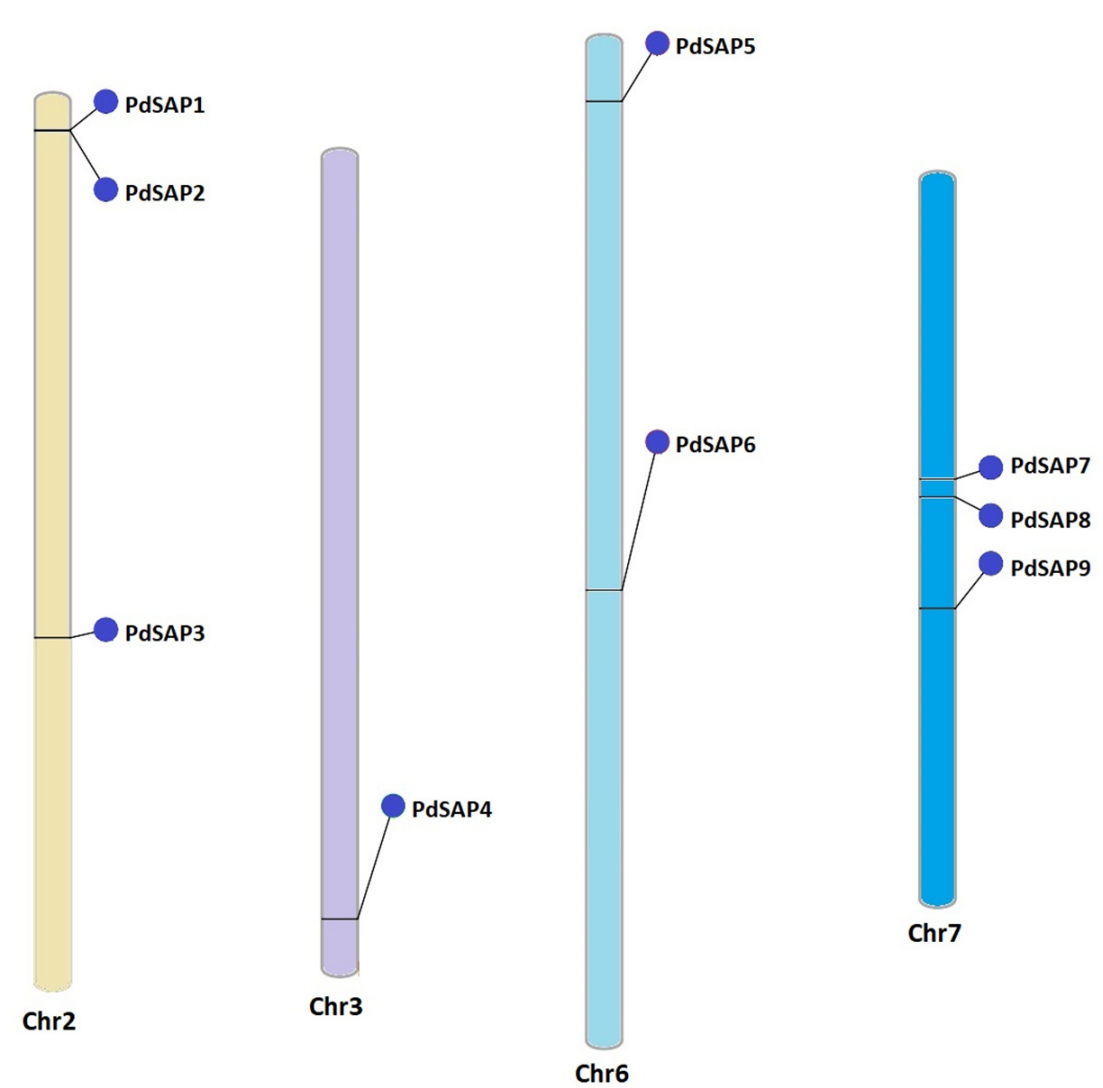
Chromosomal mapping of *PdSAP* genes. *PdSAP* genes have been mapped on the chromosomes of *P. dulcis*. Chromosome numbers are presented below each chromosome. Each chromosome is colored differently.

A phenogram predicted the chromosomal distribution of the nine SAP genes of almond. SAP genes were unevenly distributed on the chromosomes 2, 3, 6, and 7 of *P*. *dulcis* as shown in Figure 3. SAP genes were absent on the chromosomes 1, 4, 5, and 8 of almond. There were three SAP genes present on chromosomes 2 and 7, whereas chromosomes 3 and 6 contained one and two *PdSAP* genes, respectively. Chromosome 2 had only one cluster of *PdSAP* genes consisting of *PdSAP1* and *PdSAP2*. Chromosome 3 contained only one *PdSAP* gene, which was *PdSAP4*.

**Figure 4:**
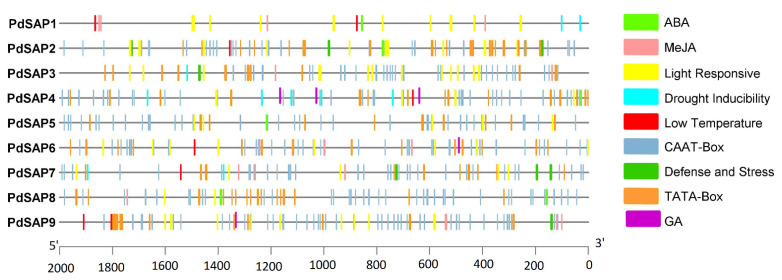
*PdSAP cis*-acting regulatory elements. Upstream promoter regions of *PdSAP* contained the *cis*-acting regulatory elements, and these are shown in various colors in the figure, with the length of the upstream region of *PdSAP* genes indicated on the *x*-axis.

A total of three hormonal and five *cis*-acting regulatory elements, namely, abscisic acid (ABA), methyl jasmonate responsiveness (MeJA), gibberellic acid (GA), drought inducibility (MYB), defense and stress (TC-rich repeat), low temperature responsive element (LTR), TATA box, and CAAT box, were selected in the upstream genomic sequences of *PdSAP*. These *cis*-regulatory elements are depicted in Figure 4. TATA box was the most prominent and the most frequently occurring compared to all other elements in all *PdSAP* sequences. *PdSAP2* contained nine ABA *cis*-regulatory elements that were higher in number as compared to the other *PdSAP* genes. CAAT box was present in all *PdSAP* sequences, containing 53 of the highest elements in *PdSAP1*. Among all, GA was the least occurring promoter element in the *PdSAP* sequences. MYB is responsible for drought inducibility with the highest number in *PdSAP7*. TC-rich elements were detected in all SAP genes that are responsible for stress responsiveness and defense. *Cis*-regulatory elements, with their start and end position in the upstream region of *PdSAP*, are provided in Data S2.

### 2.4. Domain Analyses, Motif Composition, and Gene Structure of PdSAP Genes

**Figure 5:**
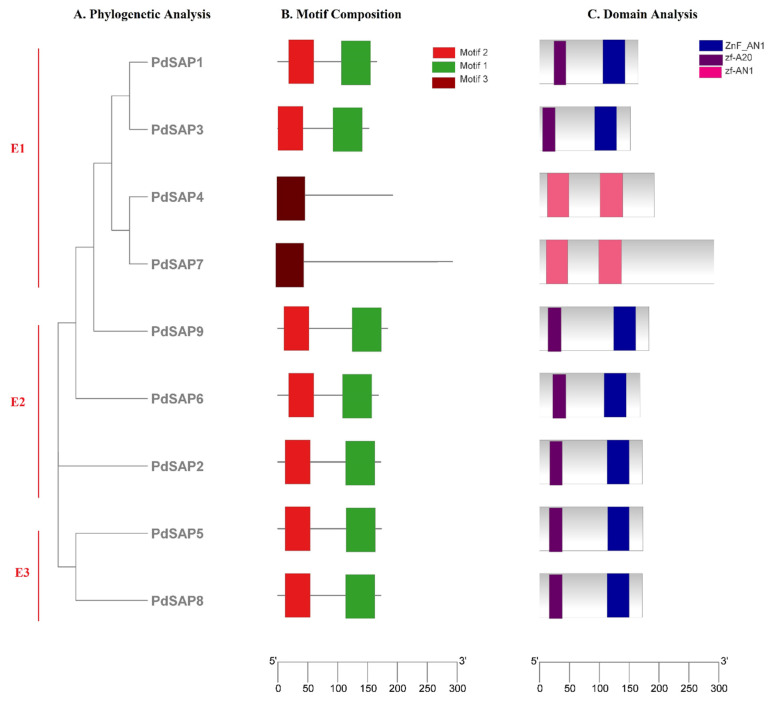
*PdSAP* motif analysis and gene structure prediction. In the figure, phylogenetic tree (**A**), motif pattern (**B**), and domains (**C**) are visualized.

**Figure 6:**
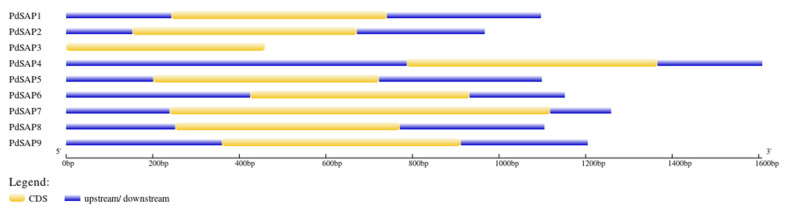
*PdSAP* gene structure prediction. Exons are in yellow. *PdSAP* genes lacked intronic regions. Blue represents the 5′ and 3′ untranslated regions (UTRs).

A phylogenetic tree of nine *PdSAP* genes was constructed consisting of three subgroups (E1–E3) as shown in Figure 5A. The E1 subgroup had four members of *PdSAP* genes, namely, *PdSAP1*, *PdSAP3*, *PdSAP4*, and *PdSAP7*. The E2 subgroup consisted of three *PdSAP* genes, namely, *PdSAP2*, *PdSAP6*, and *PdSAP9*. In the E3 subgroup, only two members of *PdSAP* genes (*PdSAP5* and *PdSAP8*) were present. Further, motif analysis of nine *PdSAP* proteins was carried out as shown in Figure 5B. Three motifs were predicted in the *PdSAP* genes, and their sequence logos are provided in Figure S1. A total of seven members of *PdSAP* genes consisted of motif 1 and motif 2, whereas motif 3 was present only in *PdSAP4* and *PdSAP7*. In seven SAP genes, both AN1 and A20 domains were present, whereas only the AN1 domain was present in the remaining two SAP genes, which were *PdSAP4* and *PdSAP7*, as depicted in Figure 5C. All *PdSAP* genes were intronless. All members of *PdSAP* genes contained UTRs at 3′ and 5′ of the genes as shown in Figure 6.

### 2.5. Gene Duplication and Synteny Analysis of PdSAP Genes

**Figure 7:**
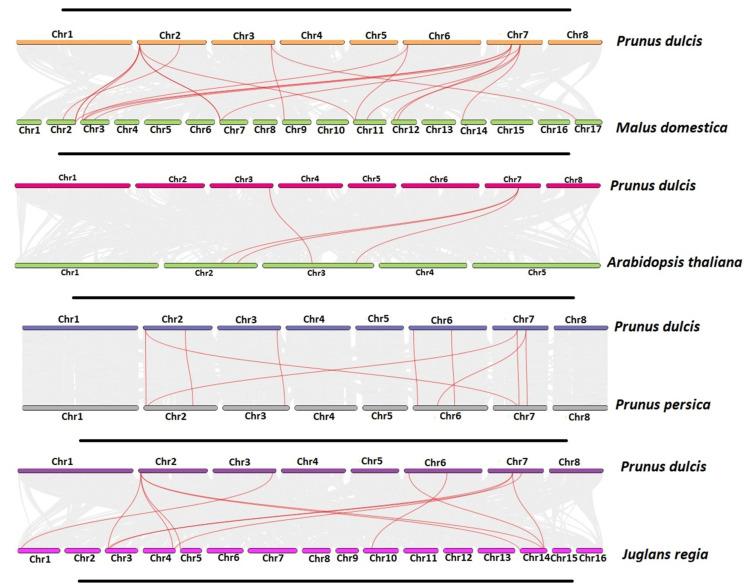
Synteny analysis of *PdSAP* genes. Synteny analysis of *PdSAP* with *M. domestica*, *A. thaliana*, *J. regia*, and *P. persica*. Red lines indicate the presence of an evolutionary relationship.

MCScanX revealed no duplication blocks in the *P. dulcis* genome. The TBTool was used to perform synteny analysis of *P. dulcis* with the genomes of *P. persica*, *A. thaliana*, *J. regia*, and *M. domestica* to analyze the presence of homologous syntenic blocks as shown in Figure 7. The synteny analysis revealed that a total of seven SAP genes in *P. dulcis* shared collinear gene pairs with *M. domestica* (19), *A. thaliana* (4), *P. persica* (10), and *J. regia* (12) as shown in Data S3. The total numbers of homologous gene pairs in *P. dulcis* with the SAPs of *M. domestica*, *A. thaliana*, *P. persica*, and *J. regia* were 15, 4, 9, and 8, respectively. Synteny maps indicated the high evolutionary homology relationship of *PdSAP* genes with SAP genes of the four other species.

### 2.6. Subcellular Localization and Physicochemical Prediction of PdSAP Genes

**Figure 8:**
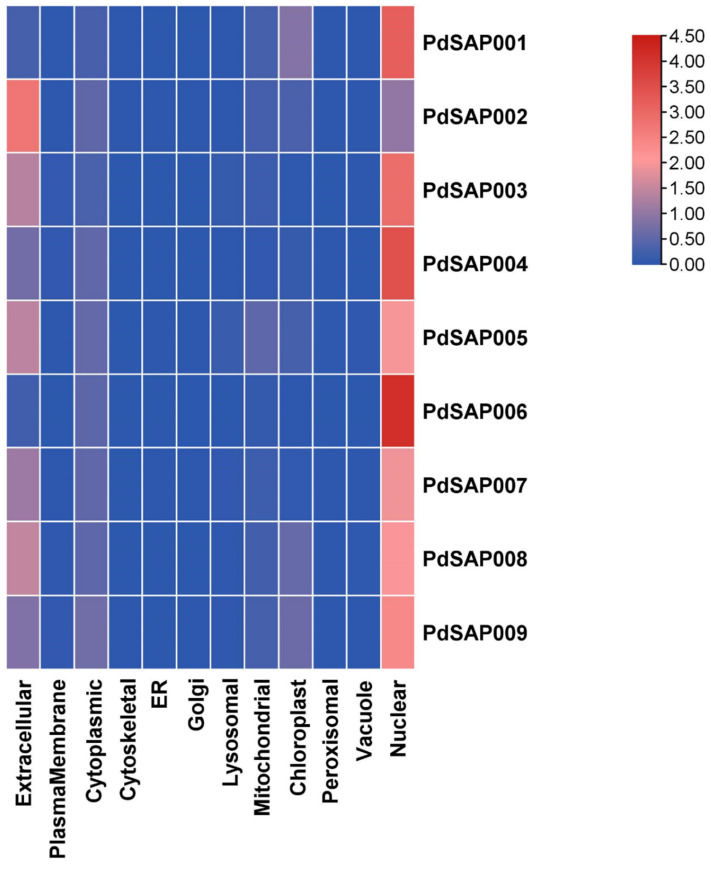
Subcellular localization of *PdSAP* gene family. Heatmap of *PdSAP* is shown. Majority of *PdSAP* genes are in nuclear region.

The molecular weight (MW) of *PdSAP* genes ranged from 16.569 kDa for *PdSAP3* to 32.124 kDa for *PdSAP7* as presented in Table S1. The isoelectric point (pI) varied from 7.46 for *PdSAP9* to 8.98 for *PdSAP4*. All of the *P. dulcis* SAP genes were found to be localized in the nucleus region, except for *PdSAP2*, which was localized in the cytoplasmic region as shown in Figure 8.

### 2.7. Gene Ontology of PdSAP Genes

**Figure 9:**
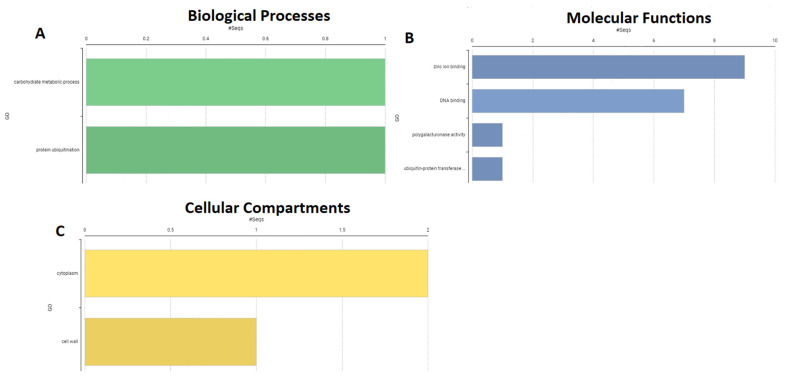
Gene ontology of *PdSAP* genes by Blast2GO. Green bars indicated biological processes (**A**), blue bars indicate molecular functions of *PdSAP* (**B**), and yellow bars indicate the presence of *PdSAP* in cellular compartments (**C**).

The molecular functions, cellular compartments, and biological processes of *PdSAP* genes were analyzed using gene ontology as presented in Figure 9. The biological processes indicated that *PdSAP* genes have a role in carbohydrate metabolism and the ubiquitination of proteins as predicted by Blast2Go. The molecular functions of *PdSAP* genes predicted their role in DNA binding and zinc ion binding, whereas the cellular compartments study revealed that the majority of *PdSAP* genes are present in the cytoplasm.

### 2.8. Differential Gene Expression of PdSAP Genes in Normal and Abnormal Fruitlets

**Figure 10:**
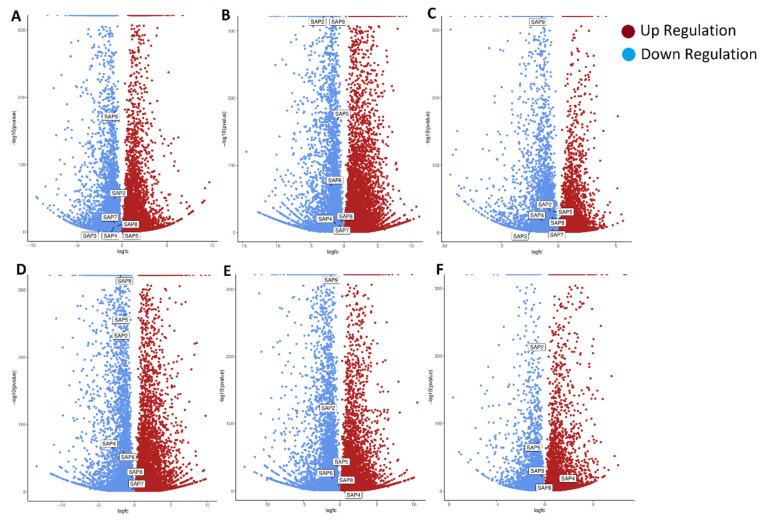
Differential gene expression of fruitlets in almonds. Volcano plots of differential expression of *PdSAP* genes is shown at developmental stage at 12 (**A**), 17 (**B**), 22 (**C**), 27 (**D**), 32 (**E**), and 37 (**F**) days after flowering in almonds fruitlets.

RNA-seq analyses of normal and abnormal fruitlets of *P. dulcis* at 12, 17, 22, 27, 32, and 37 days of development after flowering were carried out. The upregulation and downregulation of the SAP genes of normally and abnormally developed fruitlets were analyzed using DESeq2. The DESeq2 results are provided in Data S4. The DESeq2 results for only the *PdSAP* genes are presented in Data S5. Volcano plots with differential expression are presented in Figure 10. Individual volcano plots of *PdSAP* expression at 12, 17, 22, 27, 32, and 37 days of development are provided in Figures S2–S7. At day 12 of *P. dulcis*, *PdSAP2*, *PdSAP3*, *PdSAP4*, *PdSAP5*, *PdSAP7*, *PdSAP8*, and *PdSAP9* were downregulated. Similarly, *PdSAP2*, *PdSAP4*, *PdSAP5*, *PdSAP6*, *PdSAP7*, *PdSAP8*, and *PdSAP9* were downregulated at day 17. *PdSAP2*, *PdSAP3*, *PdSAP4*, *PdSAP5*, *PdSAP7*, *PdSAP8*, and *PdSAP9* also had downregulated expression at day 22 of development after flowering. *PdSAP2*, *PdSAP4*, *PdSAP5*, *PdSAP6*, *PdSAP7*, *PdSAP8*, and *PdSAP9* had downregulated expression at day 27. At days 12, 17, 22, and 27, no *PdSAP* gene had upregulated expression. *PdSAP2*, *PdSAP5*, *PdSAP6*, *PdSAP8*, and *PdSAP9* had downregulated expression at day 32 of development, whereas *PdSAP4* had upregulated expression at day 32. Similarly, at day 37 after flowering, *PdSAP2*, *PdSAP5*, and *PdSAP9* had downregulated expression, whereas *PdSAP4* and *PdSAP8* had upregulated expression. *PdSAP2*, *PdSAP5*, and *PdSAP9* had downregulated expression at 12, 17, 22, 27, 32, and 37 days of development, whereas the expression of the other *PdSAP* genes was variable.

## 3. Discussion

Almonds are consumed worldwide, as they are a nutrition-rich source of magnesium, potassium, and vitamin E. Given their importance, SAP TFs are involved in the response to abiotic and biotic stresses, in addition to cell wall synthesis [33]. The SAP gene family has been reported in various plant species but not in *P. dulcis*. In this study, we identified nine *PdSAP* genes in the genome of *P. dulcis*. A high diversity of SAP genes has been reported among different plant species, ranging from 11 members in *Zea mays* to 57 members in *Brassica napus* [34,35]. All *PdSAP* genes were mapped to four out of eight chromosomes, and no SAP genes were found on chromosomes 1, 4, 5, and 8. Similarly, twelve *CsSAP* genes were present on chromosome 6, whereas chromosome 2 lacked *CsSAP* genes in cucumber [23]. *PdSAP* genes in almonds were found to be spread out on chromosomes 2, 3, 6, and 7. In addition, one cluster of *PdSAP1* and *PdSAP2* genes was found on chromosome 2. Similarly, three *CsSAP* genes were spread out on chromosomes 1, 3, 4, 5, 6, and 7, and six *CsSAP* genes were clustered on three chromosomes in cucumber [23]. In *Medicago truncatula*, chromosome 2 contained three *MtSAP* genes, and two *MtSAP* genes were spread out on chromosomes 1, 3, and 4, each [36]. Various defense and stress responsive regulatory factors were found in the upstream promoter region of *P. dulcis*, confirming the involvement of *PdSAP* genes in stress responses. In cucumber, the same *cis*-regulatory elements were found, indicating their role in stress responses [23]. Similarly, in the *MtSAP* genes of *Medicago truncatula*, ABRE, MBS, ARE, and TGA elements were predicted [36].

The phylogenetic tree of *PdSAP* genes was divided into six subgroups with respect to *A. thaliana* and *Oryza sativa*. The G-1 subgroup was the largest group with 10 members, including *PdSAP4* and *PdSAP7* genes with the zf-AN1 domain. Subgroups G-2 and G-3 harbored *PdSAP2*, *PdSAP5*, *PdSAP6*, *PdSAP8*, and *PdSAP9* with both A20 and AN1 domains, respectively. Subgroups G-4 and G-5 contained *PdSAP3* and *PdSAP1* with both zinc finger domains. Subgroup G-6 had no *PdSAP* genes. Similarly, the phylogenetic tree of cucumber contained the seven subgroups, where group f contained two AN1 domains and groups a, b, c, d, and e contained both AN1 and A20 domains [23]. SAP genes in *Solanum lycopersicum*, *A. thaliana*, and *O. sativa* were divided into five groups [3]. The phylogenetic tree of the SAPs belonging to *Ricinus communis*, *A. thaliana*, *Jatropha curcas*, *Manihot esculenta,* and *Hevea brasiliensis* was divided into eight subgroups [37]. In addition, *PdSAP* genes within the same group shared a similar pattern of conserved domains, indicating that they may share similar functions. A conserved motif distribution pattern was observed in almond SAP proteins based on an evolutionary relationship. Three conserved motifs were identified, and these were distributed on *PdSAP* proteins, exhibiting a strong evolutionary relationship. Similar to *P. dulcis* motifs, *CsSAP5* and *CsSAP6* lacking the Znf-A20 domain also had different conserved motifs as compared to the genes containing both Znf-A20 and Znf-AN1 domains [23].

Variation in the number of exons and introns plays a pivotal role in the evolution of transcription factor protein families. Intronless SAP genes are responsible for immediate response by reducing post-transcriptional processing [38]. In the rice genome, six *OsSAP* genes had a single intron, whereas one *OsSAP8* gene had two introns. However, all the remaining *OsSAP* genes were intronless [21]. In cucumber, 9 out of 12 SAP genes were intronless [23]. In the present study, *PdSAP* genes are also intronless, indicating their immediate expression during stress. No gene duplication was observed in the *PdSAP* genes of *P. dulcis*. However, in *Medicago truncatula*, two segmental duplications were identified that were between *MtSAP2* and *MtSAP9* in addition to *MtSAP4* and *MtSAP13* [36].

*P. dulcis* SAP genes had more synteny with *P. persica* as compared to *M. domestica*, *J. regia*, and *Arabidopsis thaliana*. However, *PdSAP* genes had syntenic relationships with multiple *M. domestica* SAP genes, as *M. domestica* has 14 SAP genes. Similarly, *PdSAP* genes were also homologous to multiple *J. regia* genes, whereas only two *PdSAP* genes were homologous to four *A. thaliana* SAP genes. Most of the *P. dulcis* SAP genes were predicted to be in the nuclear region; however, two SAPs were predicted to be in the cytoplasmic region. Previously, it had been reported that SAP genes play an important role in regulating a variety of abiotic and biotic stresses, which is because of their nuclear localization in the cell [30,39]. Similarly, SAPs were predicted to be in the cytoplasm, nucleus, and endoplasmic reticulum in *Brassica napus* [40]. These localization sites were also predicted for cotton [24].

Sequence read archives (SRAs) were obtained from a study based on profiling of carbon signaling genes by Guo et al. 2021 [32]. In this study, the sequence read archives of normal and abnormal fruitlets were used to check the differential expression of *PdSAP* genes on days 12, 17, 22, 27, 32, and 37 after flowering. All *PdSAP* genes had significant expression in the normal and abnormal fruitlets. Similarly, *CsSAP1*, *CsSAP9*, *CsSAP11*, *CsSAP5*, *CsSAP7*, and *CsSAP12* had significant expression in cucumber at different stages of fruit development [23]. Some of the genes, such as *PdSAP2*, *PdSAP5*, and *PdSAP9*, had downregulated expression throughout the developmental stages, which were tested using RNA-seq. The downregulation of *PdSAP* genes suggests their expression in abnormal fruitlets. Because diapause atrophy is involved in the abnormality of fruitlets, almonds express *PdSAP* genes as a response to those stresses. However, there is no clear pattern of the upregulation of *PdSAP* genes. It is pertinent to mention that the upregulation of *PdSAP4* and *PdSAP8* on the 37th day of development suggests their involvement in fruit development at the final stages. Similar to this study, SAP expression was observed in response to NaCl and PEG stresses in *Brassica napus* [40]. In cotton, a differential expression pattern of SAP genes was reported under methyl jasmonate (MeJA), NaCl, and PEG [24]. Similarly, diapause atrophy results in abnormal fruitlets, due to which *PdSAP* genes are expressed to counter this stress. This study identifies *P. dulcis* SAP genes and provides insights into their structure as well as their evolutionary relationships. It also reports the involvement of the *PdSAP* genes expression in abnormally developed fruitlets at various growth stages as compared to normally developed fruitlets in *P. dulcis*.

## 4. Materials and Methods

### 4.1. Identification of PdSAP Gene Family in Prunus dulcis

Protein FASTA file of *Prunus dulcis* was retrieved from latest genome assembly (GCF_902201215.1) available on NCBI and was used as local database to perform BLASTp. To identify SAP gene family in *Prunus dulcis*, protein sequences of annotated SAP genes of rice and *Arabidopsis* were retrieved from TIGER rice database (http://blast.jcvi.org/euk-blast/index.cgi?project=osa1, accessed on 19 August 2021) and TAIR database (https://www.arabidopsis.org/, accessed on 19 August 2021). These retrieved sequences of SAP genes were used as a query to perform the local BLASTp using BLAST+ tool with BLOSUM62 matrix and 10 as E value. Pfam (http://pfam.xfam.org/, accessed on 19 August 2021) was used to scan all BLAST hits to check the presence of zinc finger domains [41]. Sequences containing A20/AN1 were selected after removing redundant sequences with 100% similarity. Putative SAP genes were renamed according to their chromosomal position.

### 4.2. Multiple Sequence Alignment and Phylogenetic Analysis of PdSAP

Protein sequences of SAP genes of *P. dulcis* were aligned in a FASTA file. For multiple sequence alignment, ClustalW-V2.1 was used on UseGalaxy webserver [42]. AN1 and A20 domains were analyzed with hidden Markov model (HMM) profile using Pfam database. Both Znf-AN1 and Znf-A20 domains were highlighted in the alignment of almond’s SAPs sequences. For phylogenetic tree construction, multiple sequence alignment was carried out using SAP peptide sequences of almonds with *Arabidopsis thaliana* and rice using default parameters in MegaX-V10.2.4 (www.megasoftware.net, accessed on 19 September 2021). The output file of multiple sequence alignment was used to the generate phylogenetic tree using neighbor-joining method with p-distance and 1000 bootstraps replicate. Finally, phylogenetic tree was visualized and edited in iTOL webtool (https://itol.embl.de/, accessed on 19 September 2021) [42,43].

### 4.3. Chromosomal Mapping and Cis-Acting Regulatory Analysis of PdSAP

Chromosomal positions of the SAP gene family were analyzed using phenogram webserver (http://visualization.ritchielab.org/phenograms/plot, accessed on 30 October 2021) [44]. *Cis*-acting regulatory elements and 2000 bp of genomic sequences upstream of SAP genes were retrieved using TBTool. PlantCARE database (http://bioinformatics.psb.ugent.be/webtools/plantcare/html/, accessed on 30 October 2021) was used for the prediction of *cis*-regulatory elements in the promoter region. TBTool (https://github.com/CJ-Chen/TBtools, accessed on 30 October 2021) was used to visualize *cis*-acting regulatory elements in the upstream sequences of the SAPs genes.

### 4.4. Gene Structure and Motif Analyses of PdSAP

For gene structure analysis of SAP genes of almonds, CDS and gene sequences of *PdSAP* genes were used in Gene Structure Display Server (http://gsds.gao-lab.org, accessed on 3 November 2021) [45]. Phylogenetic tree was generated using neighbor-joining method with 1000 bootstraps by MEGAX-V10.2.4 Newick. Protein domains in SAP gene sequences were analyzed in conserved domain database (https://www.ncbi.nlm.nih.gov/Structure/cdd/wrpsb.cgi, accessed on 3 November 2021). Motifs were identified using the MEME webserver (https://meme-suite.org/meme/, accessed on 3 November 2021) in the *PdSAP* genes. The output files from these servers and Newick file were used in TBTools to generate gene motif and domain illustration in SAP gene family [46].

### 4.5. Gene Duplication and Synteny Analyses of PdSAP

For gene duplication in SAP genes, MCScanX with default parameters was used [47]. BLASTp output was used in MCScanX to predict duplication events in the genome. For synteny analysis, genomic FASTA files and genomic feature files (.gff files) of *Prunus dulcis* (almonds), *Malus domestica* (apple), *Prunus persica* (peach), *Arabidopsis thaliana*, and *Juglans regia* (walnut) were downloaded from NCBI genome database and used as input files in one-step MCScanX of TBTools. The output files of one-step MCScanX were further used in dual synteny plotter for visualization of syntenic blocks [46].

### 4.6. Physiochemical Properties and Subcellular Localization of PdSAP

Physiochemical properties of SAP genes were predicted using ProtParam in Expasy webserver. It depicts structural and functional values of given protein sequences. Molecular weight and isoelectric points for SAP family were predicted using the ProtParam [48]. For prediction of subcellular localization of SAP genes, WoLF PSORT (https://wolfpsort.hgc.jp/, accessed on 30 August 2021) and Cello (http://cello.life.nctu.edu.tw/, accessed on 30 August 2021) were used [49]. WoLF PSORT predicts subcellular localization by functional motifs and amino acid composition [50].

### 4.7. Gene Ontology Analysis of PdSAP

Blast2Go was used to carry out annotation of SAP genes. Cellular compartments, biological activities, and molecular functions of SAP genes were predicted in Blast2Go. CDS sequences of *PdSAP* genes were used to perform BLASTx, InterPro Scan, annotation, and mapping. For gene ontology analysis of *PdSAP* genes, default settings of Blast2Go were used [51].

### 4.8. Differential Gene Expression of PdSAP in Fruits

RNA-sequencing data of almond ZhiPi cultivate (sequenced by Illumina hiseq 2500) were retrieved from NCBI GEO database (Biosample: SAMN12855948). Thirty plants were randomly selected based on plants’ phenotypes by Guo et al. 2021 [32]. They selected the plants with diapause atrophic growth as abnormal fruits. Samples for RNA sequencing were taken at 12, 17, 22, 27, 32, and 37 days of development after flowering by Guo et al. 2021. SRA and their accessions, along with the phenotypic traits of sampled plants, are presented in Data S6. UseGalaxy webserver (https://usegalaxy.org/, accessed on 23 August 2021) was used to convert sequence read archives (SRAs) into FASTQ files with forward and reverse reads. At each step, quality of reads was checked using FASTQC [52]. For removal of adapter and low-quality sequences from each read, Cutadapt was used [53]. To remove small and low-quality reads, quality cut-off value and minimum length of sequences were set at 20. AlmondV2 assembly (Refseq: GCF_902201215.1) was selected to align the reads in RNA STAR [54]. FeatureCounts was used to determine the number of reads per gene [55]. Normalization and differential gene expression were performed using DESeq2 tool [56,57]. Insignificantly expressed genes were filtered out at the significant adjusted *p*-value, which was set at 0.05. DESeq2 data were used to create the volcano plots for visualization of differentially expressed genes. Finally, *PdSAP* genes with differential expression were highlighted in the volcano plots. Galaxy webserver was used for all these analyses [57].

## 5. Conclusions

This study carried out identification and a comprehensive RNA sequence analysis of SAP genes in normal and abnormal fruitlets in *Prunus dulcis*. Their evolutionary relationships were established with *Arabidopsis thaliana* and *Oryza sativa*. Based on genome sequence accessibility and phylogenetic analysis, nine SAP genes were identified in *P. dulcis*, containing A20/AN1 zinc finger domains unevenly distributed on four chromosomes. Based on the multiple sequence alignment and the phylogenetic tree, *PdSAP* genes were classified into six subgroups (G1–G6) corresponding to the presence of previously reported finger domains in cucumber. Gene structure and conserved motifs had a clear pattern in each subgroup. No gene duplication events were observed. Synteny analysis revealed that the almonds were homologous to *P. persica*. Furthermore, subcellular localization and gene ontology annotation revealed the presence of *PdSAP* genes in the nucleus and the function of SAP genes. This study used RNA sequence analysis and revealed the expression of SAP genes in abnormal fruits during fruit development.

## Data Availability

The data presented in this study are available in the insert article and Supplementary Materials.

## Supplementary Materials

The following supporting information can be downloaded at: https://www.mdpi.com/article/10.3390/plants11010117/s1, Supplementary Data S1. Protein sequences of newly identified SAP genes of *Prunus dulcis* are provided. Supplementary Data S2. *Cis*-regulatory elements in upstream region of *PdSAP* genes are given. Their start and end positions are also given. Supplementary Data S3. One-to-one orthologous relationships of SAP genes with *M. domestica*, *P. persica*, *J. regia*, and *A. thaliana*. Supplementary Data S4. DESeq2 results of normal vs. abnormal fruitlets developed in *P. dulcis* at 12, 17, 22, 27, 32, and 37 days of development. Supplementary Data S5. DESeq2 results of normal vs. abnormal fruitlets developed in *P. dulcis* at 12, 17, 22, 27, 32, and 37 days of development for only SAP genes with significant expression. Supplementary Data S6. Accession of sequence read archives (SRAs) used for the RNA-seq analysis are provided. Supplementary Table S1. Name of newly identified genes, along with their accession, molecular weight, isoelectric point, and chromosome numbers, are provided. Supplementary Figure S1. Motif pattern of the three predicted motifs in the *PdSAP* genes. Supplementary Figure S2. Differential gene expression of fruitlets in almonds. Volcano plots of *PdSAP* expression in *P. dulcis* fruitlet are shown at day 12 after flowering. Supplementary Figure S3. DEG of SAP in fruitlets in *P. dulcis*. Volcano plots of DEG of *PdSAP* in fruitlets of *P. dulcis* are shown at day 17 after flowering. Supplementary Figure S4. DEG of fruitlets in almonds. Volcano plots of DEG of SAP in fruitlet of *P. dulcis* are shown at day 22 after flowering. Supplementary Figure S5. DEG of fruitlets in *P. dulcis*. Volcano plots of DEG of *PdSAP* in fruitlets of *P. dulcis* are shown at developmental stage day 27 after flowering. Supplementary Figure S6. Differential gene expression of fruitlets in almonds. Volcano plots of *PdSAP* expression in fruitlet of *P. dulcis* are shown at day 32 after flowering. Supplementary Figure S7. Differential gene expression of fruitlets in almonds. Volcano plots of differential gene expression of *PdSAP* in almond’s fruitlet are shown at day 37 after flowering.
