# Supplementary figures and images for: Genome-Wide Identification of Stress-Associated Proteins (SAPs) Encoding A20/AN1 Zinc Finger in Almond (Prunus dulcis) and Their Differential Expression during Fruit Development
Source: Plants (Basel). 2021 Dec 31;11(1):117. doi: 10.3390/plants11010117 (PMC8747467; doi:10.3390/plants11010117)

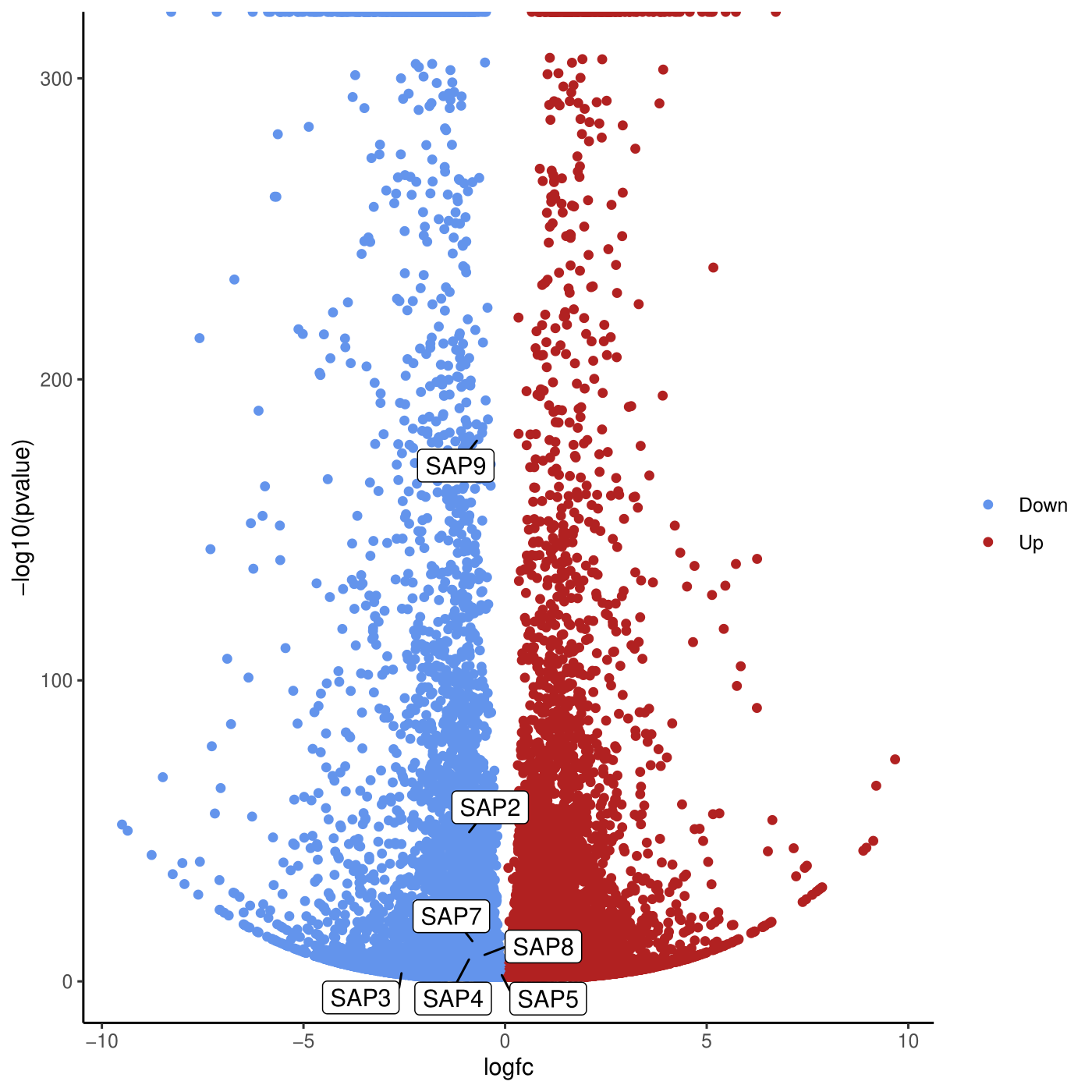

Supplement: Supplementary file 1 [file plants-11-00117-s001.zip › Supplementary Figure S2.tiff]

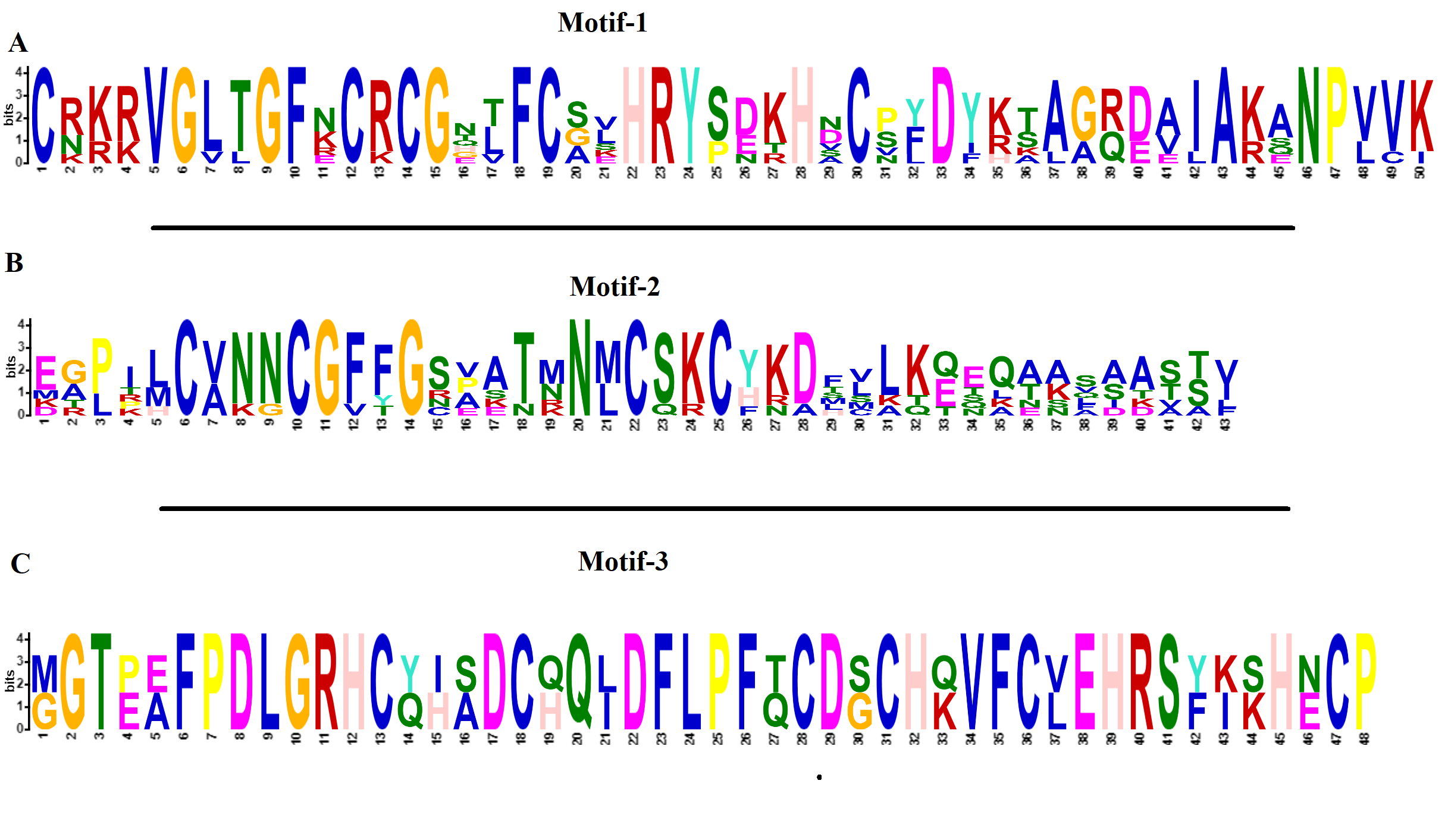

Supplement: Supplementary file 1 [file plants-11-00117-s001.zip › Supplementary Figure S1.tif]

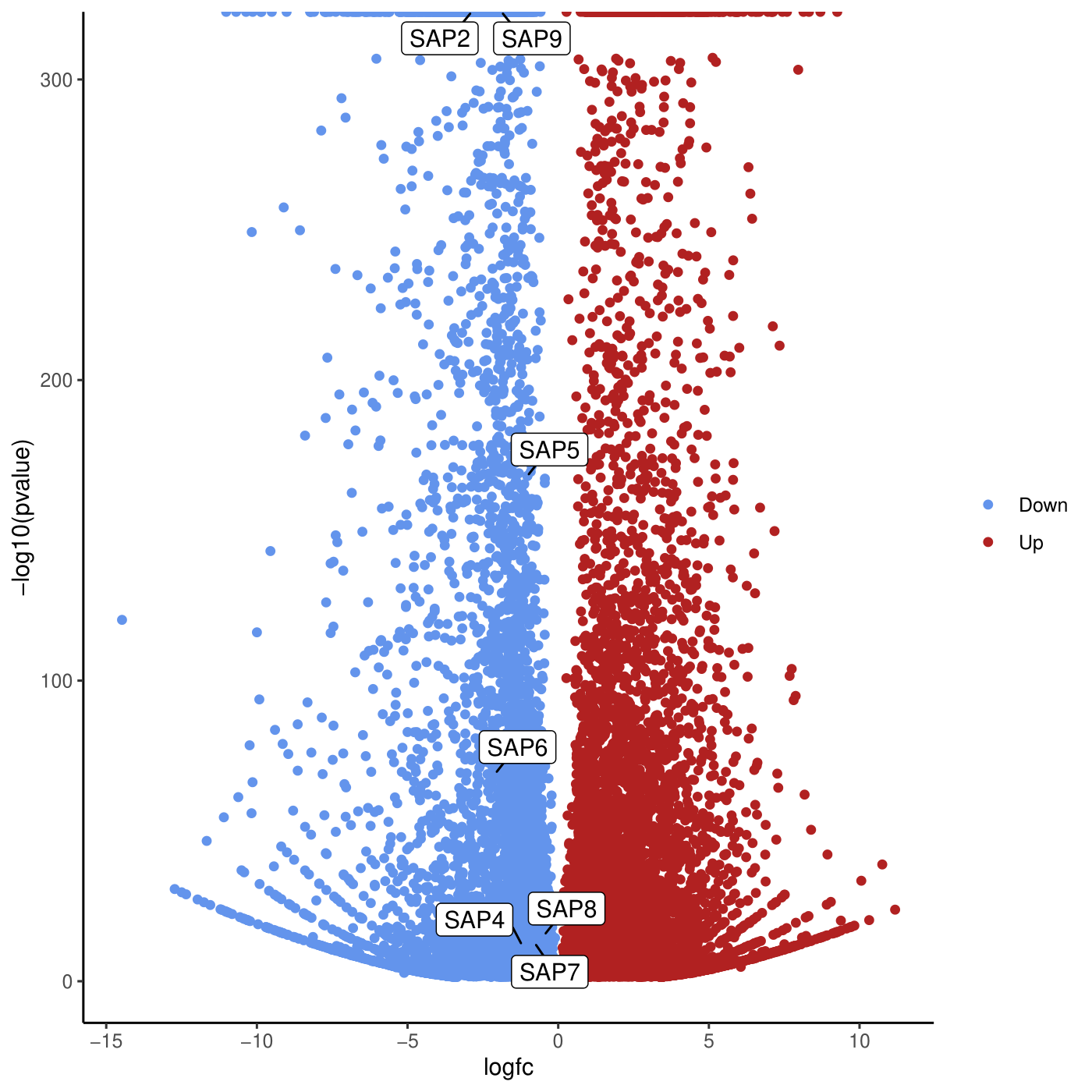

Supplement: Supplementary file 1 [file plants-11-00117-s001.zip › Supplementary Figure S3.tiff]

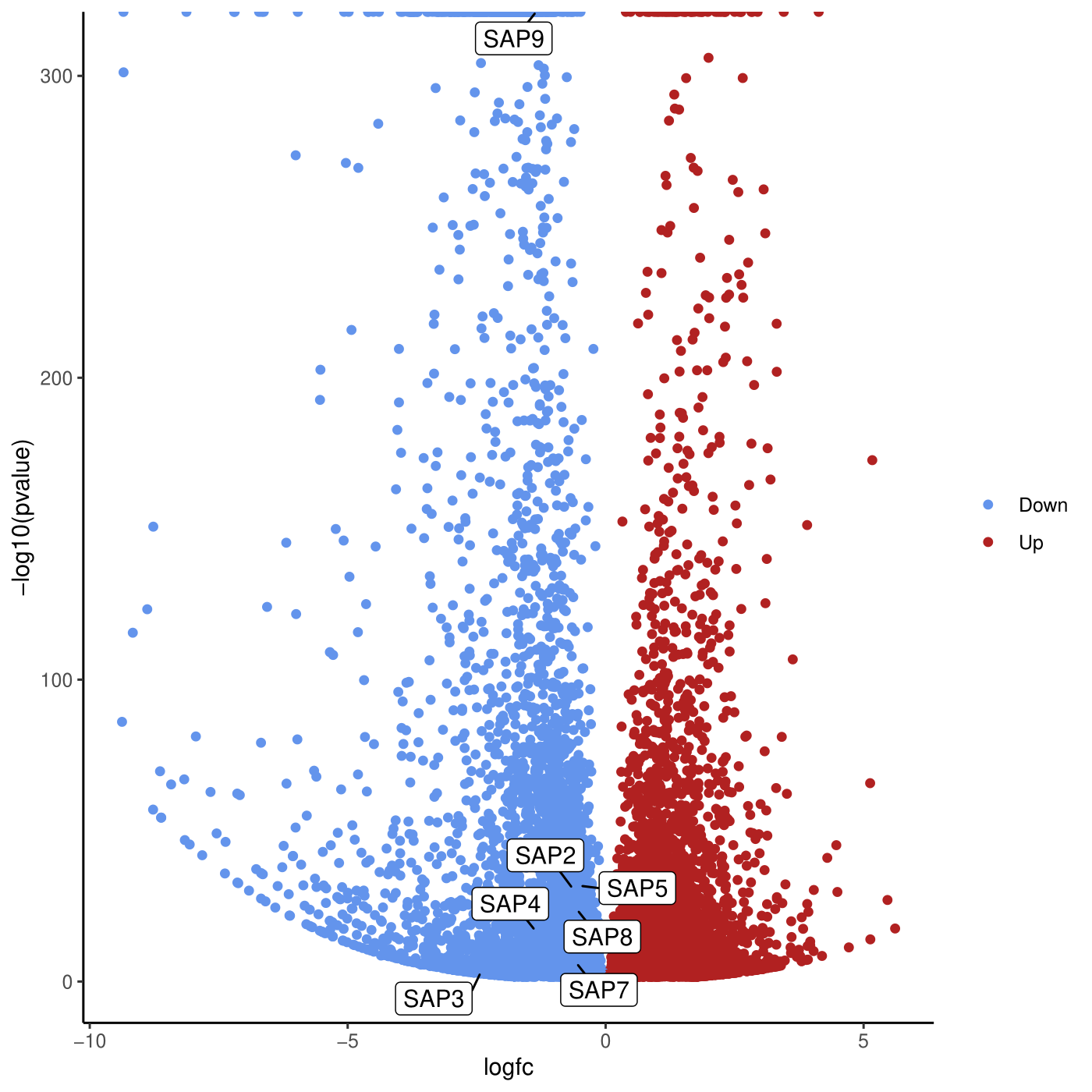

Supplement: Supplementary file 1 [file plants-11-00117-s001.zip › supplementary Figure S4.tiff]

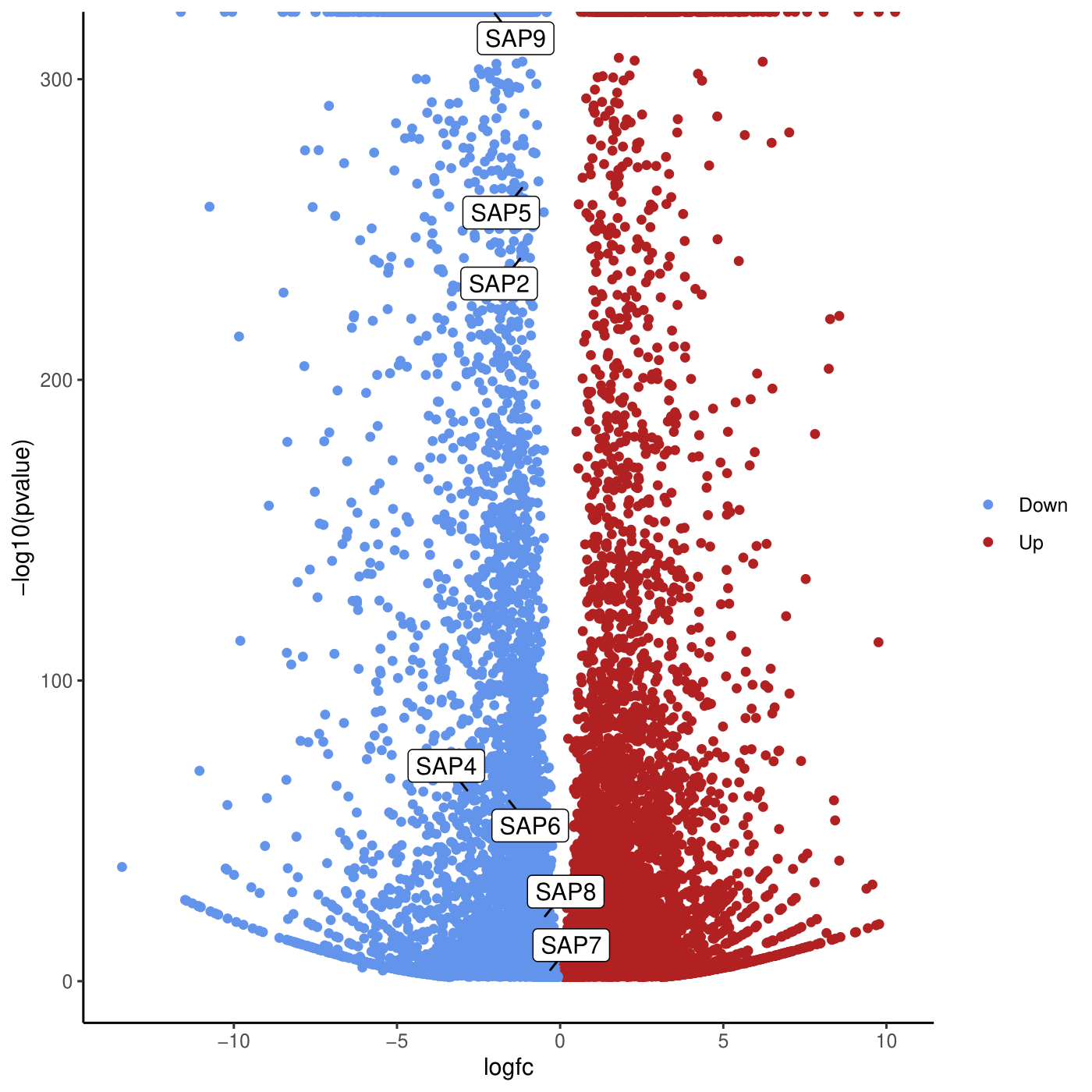

Supplement: Supplementary file 1 [file plants-11-00117-s001.zip › Supplementary Figure S5.tiff]

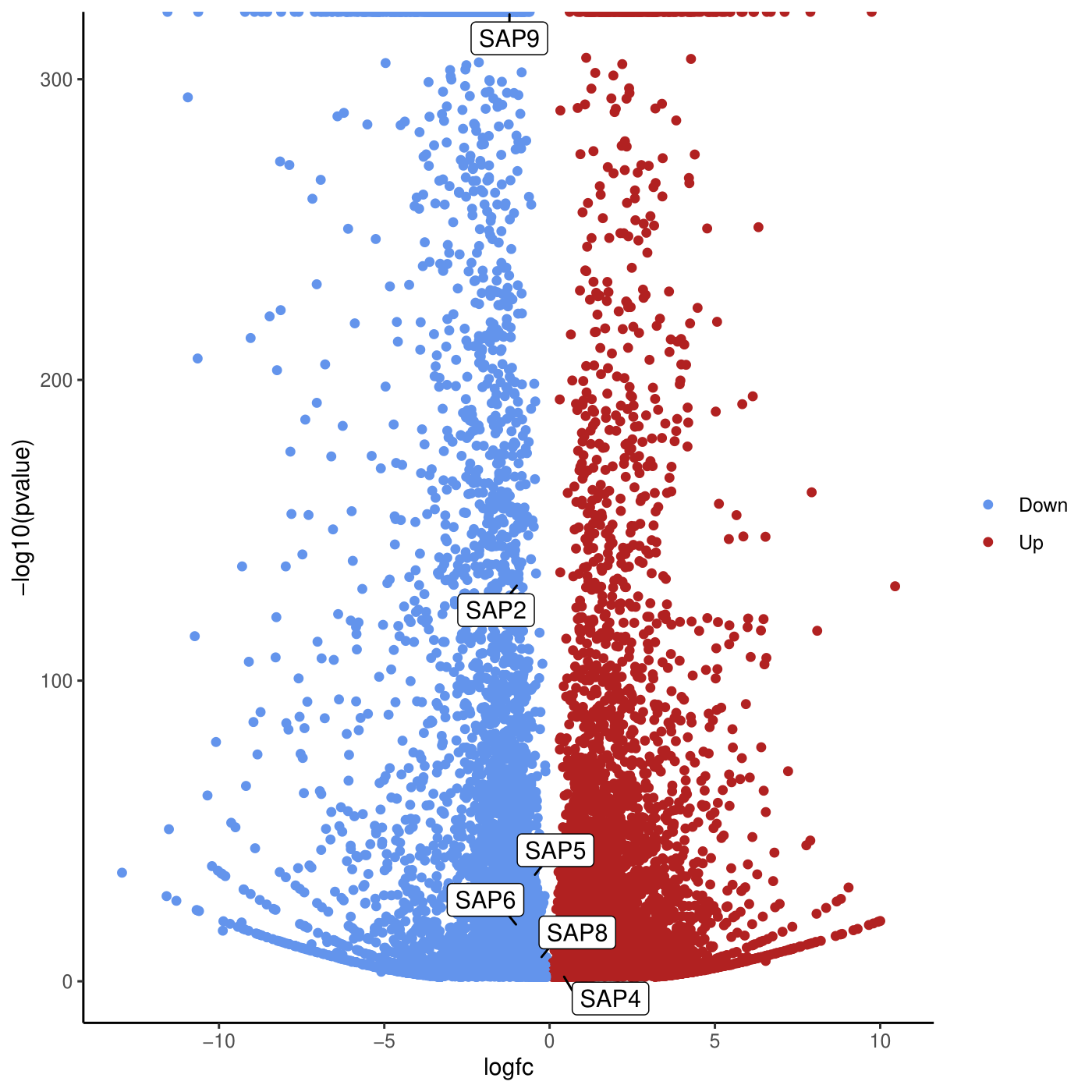

Supplement: Supplementary file 1 [file plants-11-00117-s001.zip › Supplementary Figure S6.tiff]

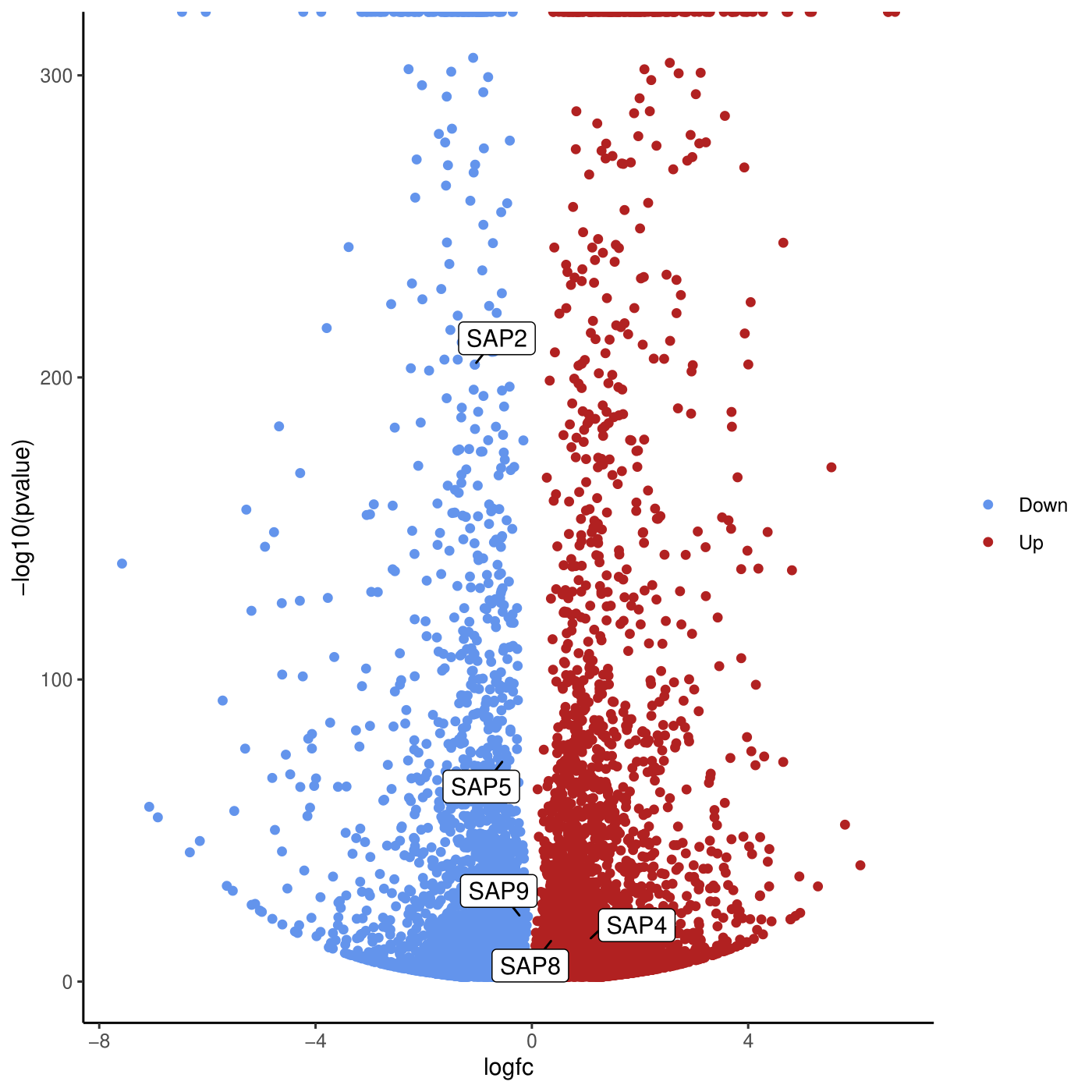

Supplement: Supplementary file 1 [file plants-11-00117-s001.zip › Supplementary Figure S7.tiff]
